# Tailored Oxidation Barrier Coatings for Mo-Hf-B and Mo-Zr-B Alloys

**DOI:** 10.3390/ma12142215

**Published:** 2019-07-10

**Authors:** Iryna Smokovych, Volodymyr Bolbut, Manja Krüger, Michael Scheffler

**Affiliations:** 1 Institute of Materials and Joining Technology, Otto-von-Guericke University Magdeburg, Universitätsplatz, 239106 Magdeburg, Germany; 2 Institute of Energy and Climate Research IEK-2, Forschungszentrum Jülich, 52425 Jülich, Germany

**Keywords:** polymer-derived ceramics, preceramic polymers, perhydropolysilazane, oxidation barrier coatings, Mo-Hf-B, Mo-Zr-B

## Abstract

The cyclic oxidation response of Mo-14Hf-23B and Mo-14.8Zr-26B (compositions in at. %) was investigated in air at 800 °C, which is a critical temperature for Mo-based alloys because of the pesting phenomenon. Rapid oxidation was observed for the unprotected samples, and an oxidation protection coating was developed based on a preceramic polymer with silicon and boron as particulate fillers. Cyclic oxidation tests of the coated samples showed excellent oxidation protection: no Mo, Hf or Zr oxides were found after testing and a small mass gain in the initial stage of oxidation indicated the formation of a glassy protection layer on the alloys surfaces after exposure to air at 800 °C.

## 1. Introduction

One of the challenging tasks in materials science is the development of novel high-temperature materials; characteristics of high temperature structural materials, besides high melting temperatures, are a high creep resistance, an appropriate tolerance for crack initiation and crack growth, resistance against oxidative, corrosive and erosive attacks, and thermal shocks [1,2]. The properties of known high-temperature structural materials can limit the advance of machine design in airplane engines or power plants [3,4]. For example, the most common materials in high-pressure turbines of aero-engines and gas turbines—nickel-based super alloys—have reached their technological limit, and are not capable of having structural components at temperatures above 1150 °C [5]. High melting temperature metals such as molybdenum alloys represent an alternative, but they suffer from rapid oxidation and a high creep rate at high temperatures [3]. The reinforcement of the refractory metals with a stronger phase, which may preferably be oxidation resistant, might solve this problem [4]. In this context, refractory metal borides, carbides and silicides are the most appropriate candidates.

Potential examples of such materials are the novel Mo-Hf-B and Mo-Zr-B alloys [6,7]. These alloys showed excellent creep properties; their creep rates are several orders of magnitude lower compared to that of a nickel based single-crystal CMSX-10 alloy [8]. Mo-Hf-B and Mo-Zr-B alloys provide also high microhardness (>1000 HV0.3) and high fracture toughness (up to 17 MPa m^0.5^) [6,9], and are potential structural materials for ultra-high temperature applications. Nevertheless, the resistance to oxidation media in a wide temperature range is a decisive factor for these alloy’s technical applications.

Polymer-derived ceramics (PDCs) occur as a new class of highly oxidation resistive materials for individual parts or as composite coatings [10,11]. Polysiloxanes, polycarbosilanes or polysilazanes are silicon containing precursors, which can be converted into polymer-derived ceramic coatings on steel to provide thermal shock resistance, creep resistance and thermal stability [12,13]. The use of fillers reduces the shrinkage of precursor coatings during ceramics formation, which leads to an in-crease of the coating thickness and prevention of cracks [14,15]. There is limited data in the literature of polymer ceramic coatings on molybdenum substrate.

In this study, the oxidation properties of the Mo-14Hf-23B and Mo-14.8Zr-26B alloys were tested at 800 °C in air, thus, taking a temperature from the pesting regime into account. Besides, protective coatings made of a preceramic polymer and oxidation resistant filler particles were developed and applied to these alloys aimed to improve their oxidation resistance under these conditions. It should be noted that the obtained results show the first results of oxidation properties, and do not include the oxidation kinetics yet.

## 2. Materials and Methods

The materials investigated in this paper were manufactured by an arc melting process. The arc melted Mo-14Hf-23B and Mo-14.8Zr-26B (in at. %) alloys were produced from elemental flakes of Mo, Hf, Zr (all from E. WAGENER GmbH, Heimsheim, Germany) and B (HMW Hauner GmbH, Roettenbach, Germany) with a purity of 99.95%, 98.0%, 97.2% and 99.0%, respectively. Processing was carried out in a Buehler arc furnace of the type MAM 1 (Edmund Buehler GmbH, Bodelshausen, Germany) in high-purity argon atmosphere. The alloys were subsequently cooled in a copper mould. The melting process was carried out at least for five times including flipping of the samples in-between each melting step to improve the chemical homogeneity.

For the cyclic oxidation tests, the Mo-Hf-B and Mo-Zr-B alloys were cut by electrical discharge machining (EDM) into samples of 2 mm × 1.5 mm × 8 mm. Cyclic oxidation tests were performed in a convection furnace (Model L9/S17, Nabertherm, Lilienthal, Germany) at 800 °C. Interval withdrawal of samples for characterization was carried out after 10 min during the initial oxidation stage (<1 h), and in 1-h steps up to 20 h followed by 5-h steps up to 50 h, respectively. The specific mass change of the samples was measured using an analytical balance with an accuracy of 0.0001 g.

For the application of a preceramic polymer coating, a dip-coating method was carried out in a self-constructed dip coater; a withdrawal speed of 3 mm/s and a sample holding time of 10 s were applied. Prior to coating, the alloy samples were ground with SiC paper FEPA P1200 (17.3 μm to 19.3 μm), and cleaned in an acetone (80 vol.%)/ethanol (20 vol.%) mixture by ultrasonic treatment, and subsequently dried. As a preceramic polymer, a commercially available perhydropolysilazane (PHPS NN 120-20, Clariant Advanced Materials GmbH, Sulzbach, Germany,) was chosen. Silicon (Si, purity: 99.50%; Thermo Fisher GmbH, Kandel, Germany) and boron (B, purity: 95.00–97.00%; Chempur, Feinchemikalien und Forschungsbedarf GmbH, Karlsruhe, Germany) filler powders (~5 μm) were introduced into the precursor solution (20 wt.% of PHPS in dibutyl ether) in a ratio of 64 PHPS + 24 Si + 12 B, all numbers in vol.%, by vigorous stirring under air.

The as-coated samples were heated to 110 °C with a slope of 2 K/min in air for 1–1.5 h to allow crosslinking. Pyrolysis was conducted in nitrogen atmosphere in a high temperature furnace (HTRRH 70-600/18, Fa. Gero Hochtemperaturöfen GmbH & Co. KG, Neuhausen, Switzerland) for 1 h at 1000 °C peak temperature; heating and cooling rates were 3 K/min. The thickness of the coating was achieved by four times overcasting the first layer followed by crosslinking and pyrolysis after each step.

The thermal transformation with respect to weight change was analyzed with a simultaneous thermal analyzer (STA 449 F3 Jupiter, Netzsch-Gerätebau GmbH, Selb, Germany; nitrogen, 50 mL/min; heating rate was 5 K/min). The most probable chemical reactions between Mo-Zr-B alloys’ constituents and perhydropolysilazane Si–N_2_–H_2_ (O_2_ in case of moisture) were formulated. Thermodynamic calculations of the free Gibb’s enthalpy of each of these chemical reactions were carried out with the software package HSC Chemistry 7.16 (Outotec Oy, Pori, Finland). The equilibrium of the most expected phases in a closed system with maximum entropy was determined in the temperature range between 600 °C and 1600 °C using the same software package. The output data are presented in Table 1. The phase fractions as a function of the temperature and the pyrolysis atmosphere were calculated.

Phase analysis was carried out on compact samples with an X-ray diffractometer (X’PERT PRO PANalytical, Malvern Panalytical GmbH, Kassel, Germany) using Co-Kα radiation. The phase identification was carried out with the analysis software X’Pert HighScore Plus (PANalytical, Kassel, Germany). Fourier transform infrared (FT-IR) spectra of the crosslinked and pyrolyzed samples were recorded on a Bruker Optics Vertex 70v from 4000–400 cm^−1^, resolution of 4 cm^−1^, using a Platinum attenuated reflectance technique (ATR) unit.

For microstructural studies, the samples were embedded in a cold-curable epoxy resin. After manual grinding from 320 down to 1200 grit size, the specimens were finished by mechanical polishing consecutively with a 3-µm and 1-µm diamond suspension. Scanning electron microscopy investigations (HR-FESEM, Zeiss Merlin equipped with an EDS (energy dispersive X-ray spectroscopy) detector, Carl Zeiss Microscopy GmbH, Jena, Germany; and FEI DualBeam Scios, FEI Company, Hillsboro, NH, USA) were carried out with respect to microstructure characterization and element analysis.

## 3. Results and Discussion

### 3.1. Cyclic Oxidation Behavior of the Mo-14Hf-23B and Mo-14.8Zr-26B Alloys at 800 °C

**Figure 1:**
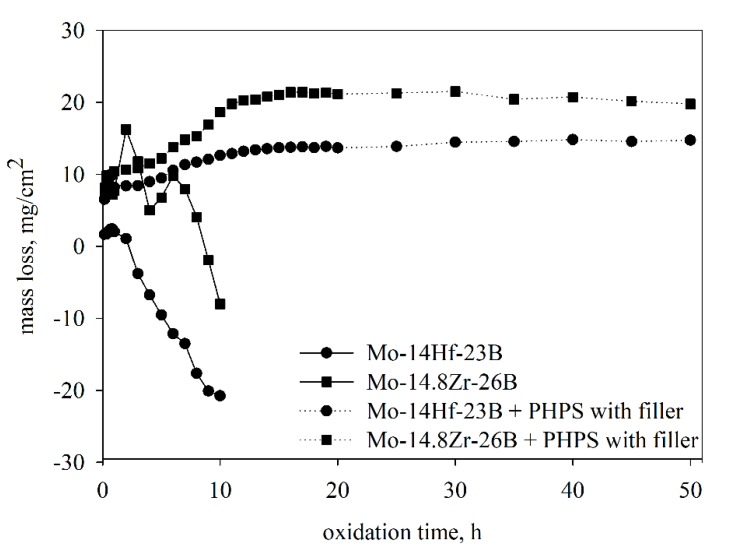
Mass change curves of uncoated and of coated Mo-14Hf-23B and Mo-14.8Zr-26B alloys under oxidation at 800 °C in air.

The oxidation behaviour of the Mo-14Hf-23B and Mo-14.8Zr-26B alloys was cyclically proven at 800 °C. This temperature was chosen since it is the harshest temperature for Mo alloys because of the occurrence of the pesting phenomenon, which means the formation of a volatile MoO_3_ phase [16]. During the tests, the samples failed catastrophically already within the first 10 h of exposure. The respective mass change curves are presented in Figure 1; mass change curves of coated samples are included for comparison and will be discussed later.

**Figure 2:**
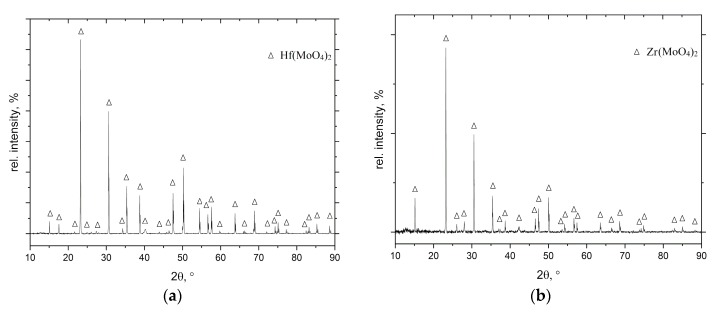
X-ray diffraction patterns of (**a**) Mo-14Hf-23B and (**b**) Mo-14.8Zr-26B alloys, both after exposure to air for 10 h at 800 °C.

At the first stage (during the first 2 h), a mass gain in both alloys was observed. This oxidation step corresponds to the formation of MoO_3_ and MoO_2_ oxides [17]. A further oxygen penetration into the samples and evaporation of MoO_3_ led to a rapid oxidation of the entire bulk of the alloys. The mass loss after 10 h of exposure in air is −21.6 mg/cm^2^ for the Mo-14Hf-23B and −9.8 mg/cm^2^ for the Mo-14.8Zr-26B alloy. After 10 h, the samples were broken due to the formation of cracks in the oxidation products. XRD (X-ray diffraction) analyses applied to the oxide surface showed the existence of Hf(MoO_4_)_2_ and Zr(MoO_4_)_2_ ternary oxides (Figure 2). A boron-containing oxide was not found by XRD investigations, which might be due to a possible amorphous structure of such oxides.

**Figure 3:**
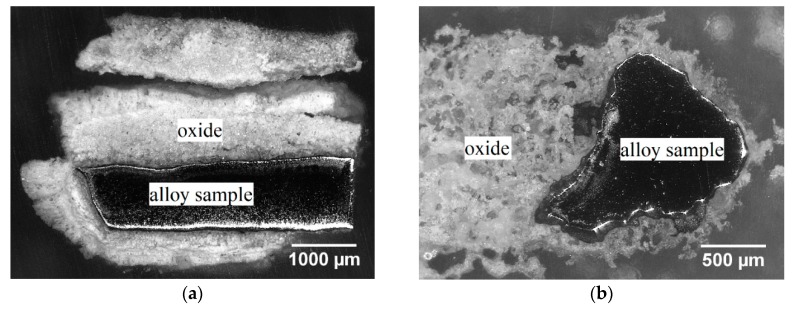
Optical microscope images of the cross-section of (**a**) Mo-14Hf-23B and (**b**) Mo-14.8Zr-26B alloys after cyclic oxidation in air at 800 °C for 10 h.

After the oxidation experiment, only a few islands of the substrate were found in the cross-sections of the samples, as shown in Figure 3.

Summarizing these first oxidation experiments, both Mo-14Hf-23B and Mo-14.8Zr-26B alloys have a poor oxidation resistance at 800 °C. This points out that the use of these alloys for high temperature applications is limited to an inert or protective atmosphere. A similar oxidation behaviour was found for other Mo-based alloys [16,17,18]: A high volume fraction of the Mo solid solution phase in Mo-Si-B alloys affects the MoO_3_ formation and its evaporation in a temperature range from 400 °C to 800 °C [17]. There is an exception for high Si-containing Mo-Si-B alloys, which form a SiO_2_-B_2_O_3_ passive glassy layer by their constituents as a result of oxygen incorporation at high temperatures [16]. A major drawback of these material concepts is that in the very first heating procedure the SiO_2_ formation is expected at temperatures above 650 °C, when the MoO_3_ evaporation has already started, and the viscosity of the glassy layer is too high to fill gaps and holes in the materials surface after MoO_3_ volatilization [18]. Therefore, the oxidation of a certain amount of material from the surface cannot be avoided.

### 3.2. Oxidation Barrier Coatings

#### 3.2.1. Thermal Analysis

A novel concept of oxidation protection of Mo-Si-B based alloys, besides other concepts [18,19,20,21], is the use of filler-loaded polymeric Si precursors and their thermal conversion into ceramic layers. In [22,23], the polymeric precursor (also addressed as preceramic polymer) perhydropolysilazane (PHPS) was part of a coating system that demonstrated high temperature oxidation resistance at 800 °C and 1100 °C up to 100 h of exposure in air. After an initial thermal treatment in N_2_ atmosphere, the PHPS coating contains mainly Si_3_N_4_ or SiO (N) amorphous ceramics and elemental Si; the latter is able to trap oxygen to form a SiO_2_ passivation layer. Passivation layer formation provides a parabolic oxidation kinetics [10,12,13]. This oxidation protection mechanism, based on a thermally stable SiO_2_ layer on the outside, might become a profitable alternative for the improvement of oxidation resistance of Mo-Hf-B and Mo-Zr-B alloys.

Following the idea to provide sufficient B_2_O_3_ to the SiO_2_ passive glassy layer for viscosity adjustment [16], Si and B particulate fillers were added to the preceramic polymer coating system; a glassy SiO_2_-B_2_O_3_ layer may have a good adherence to the substrate material, and may serve to overcome the mismatch of the coefficients of thermal expansion between coating and alloys.

For the coating system, a slurry composition of 64 PHPS + 24 Si + 12 B, all numbers in vol.%, was chosen. The resulting Si/B ratio was predetermined by experimental data [22,23], where the above-mentioned type of coating system was applied to Mo-Si-B alloys.

**Figure 4:**
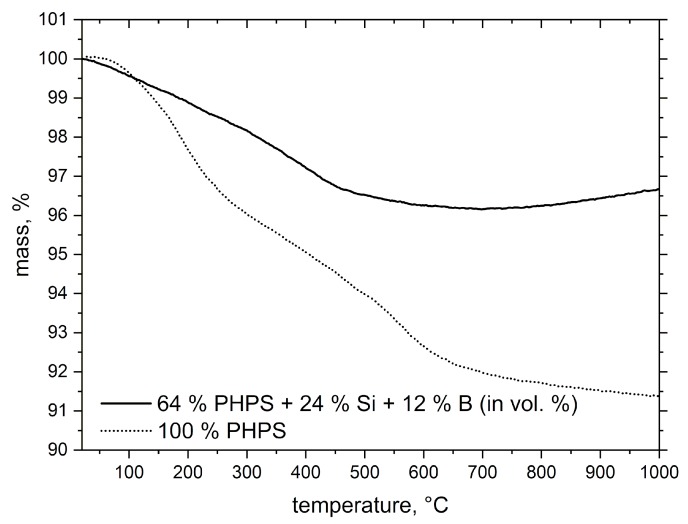
Mass change curves of crosslinked plain PHPS and PHPS + (24Si + 12 B) volume% of fillers (balance to 100 vol.% PHPS) after pyrolysis in N_2_ atmosphere.

The thermogravimetric analysis of the coating slurry is represented in Figure 4. The thermal transformation curves demonstrate significantly reduced mass loss of the particle-filled PHPS in comparison to the plain PHPS. The main mass loss of −8.6 wt.% of the previously crosslinked PHPS was observed in the temperature range up to 700 °C—this is due to the release of H_2_ and NH_3_, Equations (1) and (2), during thermally induced structural rearrangement, and the conversion of the polymer to an amorphous ceramic material, as shown in [10,12,13]:

≡Si–H + =N–H → ≡Si–N= + H_2_(g) (1)

3 ≡Si–NH–Si≡ → 2N(Si≡)_3_ + NH_3_(g) (2)

The mass loss is decreasing to −3.5 wt.% in the (24 Si + 12 B) vol.% filled system due to reduction of the PHPS content. The chemical rearrangement of the constituents of the Si_x_N_y_ ceramic in the nascent state takes place above 500 °C.

Due to the presence of hydroxyl groups on the filler’s surface [14], and the high reactivity and sensitivity of PHPS to moisture and –OH groups, siloxane bonds (*Si*–*O*–*Si*) form according to chemical reactions (3) and (4), [10]:

≡Si–OH + H–Si≡ → ≡Si–O–Si≡ + H_2_(g) (3)

≡Si–OH + NH_2_–Si≡ → ≡Si–O–Si≡ + NH_3_(g) (4)

At this stage, the nitrogen atoms are particularly replaced by oxygen atoms, thus, the formation of silicon oxide and silicon oxynitride amorphous ceramic besides of Si_3_N_4_ might be possible [22]. Günthner et al. [11,15] reported nanoscopic free Si resulting from the process of PHPS polymer-to-ceramic conversion. The interaction of this fine, particular Si with N_2_ atmosphere might result in an increase of the Si_3_N_4_ or SiNO phase amount, which explains the mass gain observed from 750 °C to 1000 °C as detected at the PHPS/filler curve (see Figure 4). The total ceramic yield of the composite after thermal treatment at 1000 °C in N_2_ is 96.6 wt.%.

To design a possible coating composition as the result of PHPS interaction with filler materials, pyrolysis atmosphere and alloy as substrate, thermodynamic calculations of the most probable chemical reactions were carried out. The ability of the reactants to interact and the directions of the chemical reactions were determined by a change of the isobaric-isothermal potential, ΔG_°*T*_ [24]. For the equilibrium calculation, the amounts of Si, N_2_, H_2_ as components of PHPS were set to 100 g of precursor (see Table 1), and Si and B as fillers were added according to their mass fraction in the crosslinked samples. The amount of Mo, Zr and B from the alloy surface was determined by “trial and error”, choosing the minimal quantity of component for the formation of expectable phases without their excess as pure elements at the phase equilibrium. The amount of O_2_ was varied in the range from 0.001 to 0.1 mol. The amount of N_2_ was varied in order to investigate its influence on the phase formation within the reaction system.

With respect to thermodynamics, the formation of Si_3_N_4_, Si_2_N_2_O and SiO_2_ may occur according to chemical reactions shown in Equations (5) and (6), (ΔG_1000 °C_ = −2648.294 kJ and ΔG_1000 °C_ = −2942.361 kJ, respectively):

Zr + Mo + 9Si + 2B + 5N_2_(g) + 2O_2_(g) → 2Si_2_N_2_O + Si_3_N_4_ + ZrO_2_ + MoSi_2_ + 2BN (5)

2Zr + 5Mo + 5Si + B + N_2_(g) + 3O_2_(g) → SiO_2_ + ZrSiO_4_ + Mo_5_Si_3_ + ZrN + BN (6)

**Figure 5:**
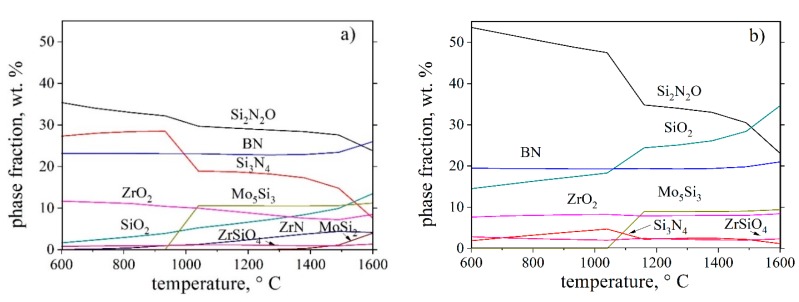
Calculated equilibrium phase amounts in the temperature range from 600 °C to 1600 °C of (**a**) Si-N_2_-H_2_-O_2_-Mo-Zr-B (0.238-0.596-0.193-**0.050**-0.082-0.019-0.036) mol and (**b**) Si-N_2_-H_2_-O_2_-Mo-Zr-B (0.238-0.596-0.193-**0.100**-0.082-0.019-0.036) mol.

Besides, at the equilibrium, other phases based on the above listed elements and their compounds are present: BN, ZrO_2_ and some amount of Mo_5_Si_3_, MoSi_2_, ZrSiO_4_ and ZrN. The presented phases were found simultaneously at the equilibrium in the investigated temperature range between 600 °C and 1600 °C, where Mo_5_Si_3_ and MoSi_2_ were found to be likely at higher temperatures, here starting from 956 °C and 1220 °C, respectively, see Figure 5. Molybdenum silicides and zirconium phases in the ceramic-metal system may be chemically compatible with the Mo-14.8Zr-26B alloy and being present in a small quantity may lead to a minimization of thermal mismatch between the substrate and the coating, thereby proving a good adhesion. On the other hand, ZrO_2_ and ZrN phases are known for their promising chemical and mechanical properties, which promulgate them as perspective candidates with high wear and corrosion resistance [25].

The main compound in the coating system is Si_2_N_2_O. In accordance with the above calculated thermodynamic systems (see Figure 5a), the amount of silicon oxynitride is 24 wt.% to 36 wt.% at temperatures between 600 °C and 1600 °C. The increase of O_2_ in the system from 0.05 mol to 0.1 mol affects the formation of both, Si_2_N_2_O and SiO_2_, (Figure 5b). In a temperature range between 1000 °C and 1100 °C, a noticeable decrease of the Si_2_N_2_O phase amount and an increase of SiO_2_ phase quantity were found. The maximal amount of Si_3_N_4_ in this system was calculated for a temperature of 1050 °C. According to the previous diagrams on the assumption of O_2_ amount, the correlation of a maximum quantity of Si_2_N_2_O and SiO_2_ protective phases and a minimum quantity of Mo_5_Si_3_ and MoSi_2_ accessory phases, which are undesired in a high amount in the coating, is evident in the temperature range of 900–1100 °C. Thus, this temperature interval should be chosen for the coating’s pyrolysis.

It was found in our calculations that an increase of the N_2_ amount within the system does not influence the equilibrium of the solid phases. From this, it was concluded that the amount of N_2_ resulting from the PHPS preceramic polymer is sufficient to form nitrogen-containing phases within these systems, and pyrolysis in argon instead of in nitrogen becomes an option.

#### 3.2.2. Phase Composition

**Figure 6:**
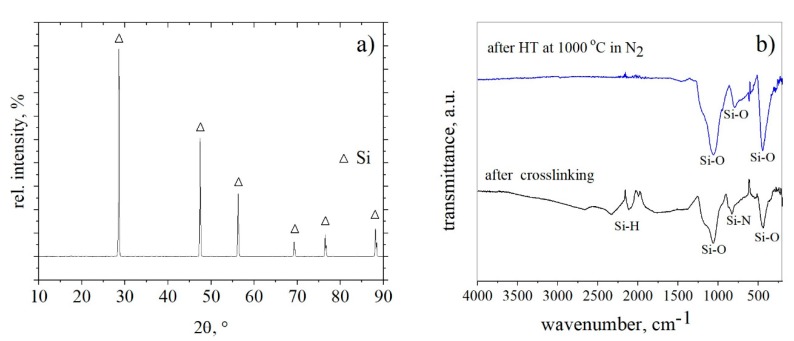
(**a**) X-ray diffraction patterns of the coating material after pyrolysis at 1000 °C in N_2_ (heating rate and cooling rate were 3 K/min and the dwell time at max temperature was 1 h); (**b**) FT-IR (Fourier-transform infrared spectroscopy) spectra of the plain, Si- and B-filled coating system after crosslinking at 110 °C in air and after pyrolysis at 1000 °C in nitrogen.

To estimate the real phase composition of the coatings after processing, XRD and FT-IR analysis were performed with the coating materials. The coating slurry was dried and crosslinked in a drying furnace at 110 °C for 1.5 h followed by grinding with a mortar and pestle to a fine powder (˂44 µm) and heat-treated in a tubular furnace in N_2_ at 1000 °C. The powders obtained after pyrolysis were subjected to X-ray diffraction analysis, (Figure 6a).

The only crystalline phase in the coating system was silicon; silicon and boron were added as fillers, (see coating composition in Materials and methods). Boron was not detected by XRD analysis because of its amorphous state.

In the FT-IR spectra, the crosslinked absorption bands were detected corresponding to Si–H stretching vibration at 2120 cm^−1^, Si–N vibrations at 840–1020 cm^−1^ and Si–O vibrations at 1063 cm^−1^ (stretching mode region) and at 456 cm^−1^ (rocking mode region) [26,27,28], (Figure 6b). This demonstrates that at the stage of crosslinking, oxygen atoms particularly replaced nitrogen atoms resulting in silicon oxynitride and silicon oxide amorphous ceramic. After pyrolysis in nitrogen at 1000 °C, the Si–H absorption band completely disappears because of the dehydrogenation reaction, and the only absorption peaks of Si–O bonds were detected in the composite. The Si–O vibrational peak at 1063 cm^−1^ was found to have a higher intensity compared to that of the crosslinked material. From this, we conclude a higher number of Si–O–Si units within the pyrolyzed material. The incorporation of nitrogen into the material cannot be excluded nor confirmed. Since no other phase than elemental silicon was found by XRD, the SiO_2_ phase is supposed to be an amorphous/glassy phase.

This coincides well with the results of Günthner et al. [11,15] as mentioned above. Free Si might act as an oxygen trap and provide a self-healing functionality due to the formation of SiO_2_. In summary, the coating system consists of an amorphous SiO_2_ phase, a SiON phase which is probably Si_2_ON_2_, elemental silicon and elemental boron.

**Figure 7:**
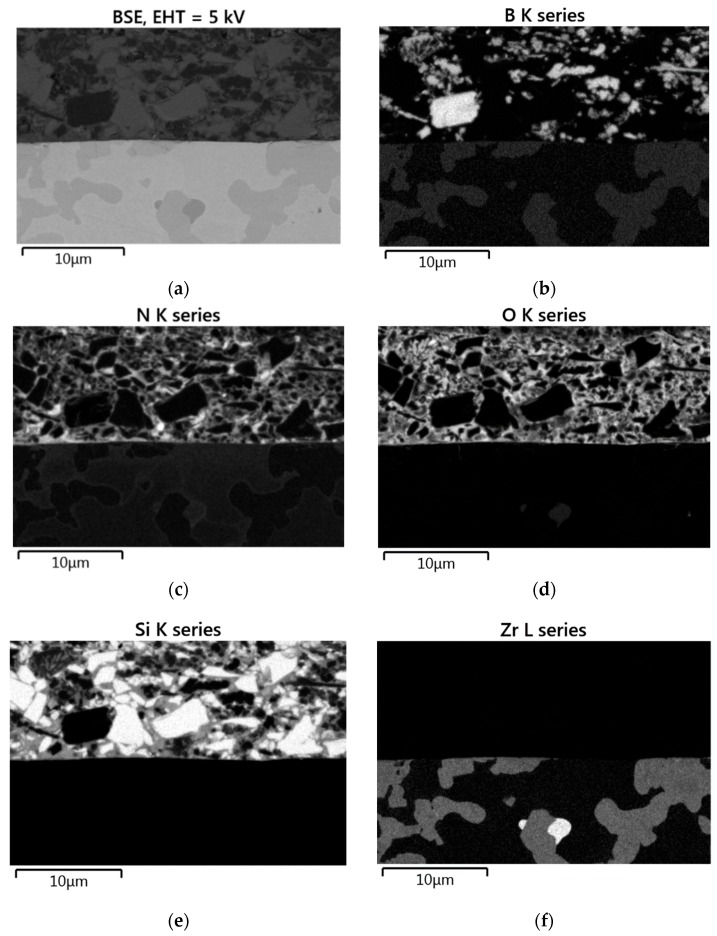
(**a**) SEM micrograph, and (**b**–**f**) EDS element mapping of a coated Mo-14.8Zr-23B alloy after pyrolysis at 1000 °C in N_2_.

In another set of experiments, real coatings on Mo-14.8Zr-23B and on Mo-14.8Zr-26B were characterized after pyrolysis in nitrogen at 1000 °C. Scanning electron microscope (SEM) analyses and EDS element mapping results are shown exemplarily for the Mo-14.8Zr-23B sample in Figure 7.

The coating layers did not show any phase segregation across the entire samples cross section, and its average thickness on the planar sample surfaces was 400 µm and 420 µm for Mo-14Hf-23B and Mo-14.8Zr-26B, respectively. However, it should be noted that the thickness of the coating was only 10 µm at the corners of the samples, while in some other regions (typically at the horizontal flat surfaces) the coating was more than 600 µm thick.

Element mappings confirmed the presence of Si, N, B and O in the coating layer. The light white particles of Si are surrounded by the PDC-matrix appearing light grey, and particularly reacted to form SiO_x_ or SiO(N) phases. The close neighborhood of boron und silicon suggests the formation of a borosilicate glassy phase. This glassy phase might have a viscosity low enough to fill gaps and cracks in the substrate material and protect it from oxidation: oxygen possesses a very small diffusion coefficient (≈1 × 10^−9^ cm^2^/s) when passing SiO_2_-B_2_O_3_ glass [29].

#### 3.2.3. Cyclic Oxidation of the Coated Samples

**Figure 8:**
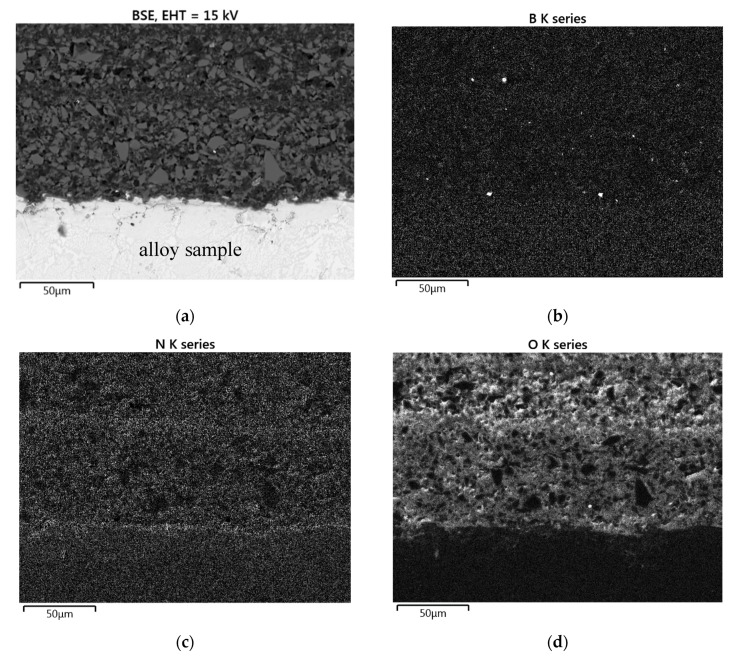

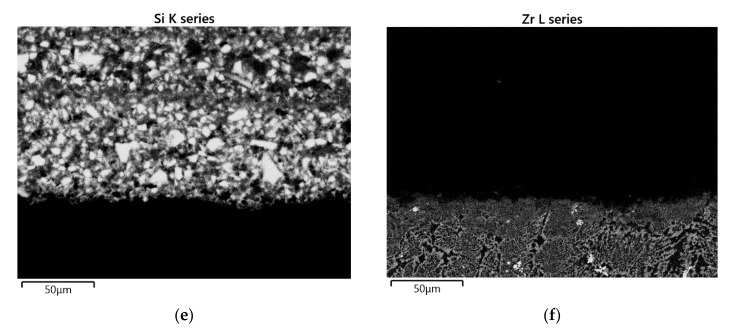
(**a**) SEM micrograph, and (**b**–**f**) EDS elements mapping of a PHPS/Si-B-coated Mo-14.8Zr-26B alloy after crosslinking, with pyrolysis at 1000 °C in nitrogen and oxidation testing at 800 °C in air.

The coated Mo-14Hf-23B and Mo-14.8Zr-26B alloys were exposed to cyclic oxidation at 800 °C in air; the corresponding mass change curves are shown in Figure 1 in comparison to the non-coated samples. During the first 10 h of the oxidation process, a mass increase of the coated samples was detected. This behaviour is opposite to the one observed for the uncoated specimens, which show a significant mass loss already at the beginning of the oxidation test. The mass gain of the coated Mo-14Hf-23B and Mo-14.8Zr-26B alloys amounts 13.2 mg/cm^2^ and 21.5 mg/cm^2^, respectively, after the first 10 h of exposure. This might be explained by the formation of a glassy oxide layer on the sample surface resulting from silicon and boron oxidation, as indicated by EDS element mappings as shown in Figure 8. After 10 h of oxidation the growth of the protective oxide layer reaches a steady state. This is indicated by stagnation in the mass gain.

Even after 50 h of oxidation testing in air at 800 °C, the distribution of Si, N, B and O in the oxidized coating layers are retained (EDS mapping results, Figure 8), and no hints to the presence of Zr or Mo oxides was found in the coating layers. This points out that no oxidation processes occur after the alloy samples were coated, crosslinked, pyrolyzed and exposed to air. However, there was a slight increase of the coating thickness after high temperature exposure to air; coating thicknesses increased to 460 µm, and 480 µm for Mo-14Hf-23B and Mo-14.8Zr-26B alloy, respectively. This is caused by a SiO_2_ surface layer formation resulting from the chemical reaction of free Si with oxygen, see Figure 8.

Thus, the coating system with PHPS + (24Si + 12B) vol.% provides an excellent protection effect against oxidation at 800 °C.

## 4. Conclusions

Cyclic oxidation tests of Mo-14Hf-23B and Mo-14.8Zr-26B alloys demonstrated a poor oxidation resistance of this type of materials at 800 °C in air. This is caused mainly by catastrophic Mo oxidation at intermediate temperatures. However, a new coating system was developed based on the preceramic polymer PHPS with Si and B fillers, which was applied to the substrate materials to protect the alloys from oxidation. After crosslinking and pyrolysis, the PHPS polymer transformed mainly into SiO_2_ and SiO (N) amorphous glasses/ceramics and dispersed elemental Si; Si is a precursor to form a dense and stable SiO_2_ coating on Mo-(Hf,Zr)-B substrates. The PHPS/Si-B coating provides excellent protection against oxidation at 800 °C in air, which is the harshest temperature for uncoated alloys of this type.

## Figures and Tables

**Table 1:** Chemical composition of the system polysilazane (PHPS NN 120-20; Si–N_2_–H_2_) alloy (Mo, Zr, B) and pyrolysis atmosphere (O_2_, N_2_) used for thermodynamic calculations.

Components Amount, mol:			
Polysilazane (PHPS NN 120-20)		From the Alloy’s Surface	From the Atmosphere
Ar			1.0
N_2_	0.596		0.200–0.400
H_2_	0.193		
Si	0.238		
Mo		0.082	
Zr		0.019	
B		0.036	
O_2_		0.001	0.010–0.100
